# Management of Steroid-Induced Glaucoma in a Patient with Pyoderma Gangrenosum

**DOI:** 10.3390/jcm12082930

**Published:** 2023-04-18

**Authors:** Ji Yeon Byun, Yong Koo Kang, Yong Hyun Jang, Young Kook Kim, Dai Woo Kim

**Affiliations:** 1 Department of Dermatology, Ewha Womans University College of Medicine, Seoul 07804, Republic of Korea; 2 Department of Ophthalmology, School of Medicine, Kyungpook National University, Daegu 41944, Republic of Korea; 3 Department of Dermatology, School of Medicine, Kyungpook National University, Daegu 41944, Republic of Korea; 4 Department of Ophthalmology, Seoul National University College of Medicine, Seoul 03080, Republic of Korea

**Keywords:** pyoderma gangrenosum, glaucoma, Ahmed glaucoma valve, microinvasive glaucoma surgery

## Abstract

Pyoderma gangrenosum (PG) is an uncommon inflammatory skin disorder typically presenting as painful skin ulcers, which may also exhibit extracutaneous findings. PG can occur at the site of trauma or surgery, which is known as the pathergic phenomenon. A 36-year-old man developed bilateral steroid-induced glaucoma after prolonged systemic immunosuppressive treatment for cutaneous pyoderma gangrenosum. After successful Ahmed glaucoma valve implantation surgery with donor scleral patch graft in the right eye, the same surgery failed repeatedly in the left eye and complicated with the prolonged conjunctival necrosis and the exposure of the donor scleral patch graft. Under the impression of ocular involvement of PG, microinvasive glaucoma surgery (MIGS) with XEN^®^ Gel Stent was performed in the left eye; the conjunctival bleb was successfully formed without conjunctival necrosis, and intraocular pressure was well maintained. Ophthalmic surgery can be complicated in patients with PG, and the surgical option should be selected prudently to minimize surgical trauma. MIGS, as a minimally invasive surgical technique, could offer an advantage for patients with PG.

## 1. Introduction

Pyoderma gangrenosum (PG) is an uncommon inflammatory skin disorder, which classically presents as painful skin ulcers. It may develop following minor trauma or surgical injury, which is known as the pathergic phenomenon [1]. PG is believed to occur in association with systemic inflammation, and many cases are associated with autoinflammatory or hematologic conditions and typically respond to immune suppression [2].

PG lesions most frequently affect the lower extremities [2]. Extracutaneous involvement in PG may occur before, during, or after the appearance of skin lesions. The most common extracutaneous manifestations of PG are in the lungs, followed by ocular and visceral involvement [3]. Ocular manifestations in PG are rare, with only several case reports in the literature. A common manifestation is necrotic ulceration and fistula in the eyelids, but there have been reports of PG involvement in the sclera, conjunctiva, cornea, and orbit [4].

Herein, we describe a patient with steroid-induced glaucoma (SIG) following prolonged immunosuppressive treatment for PG, who developed ocular involvement of PG after Ahmed glaucoma valve (AGV) implantation surgery and successfully managed with minimally invasive glaucoma surgery (MIGS).

## 2. Case Presentation

**Figure 1:**
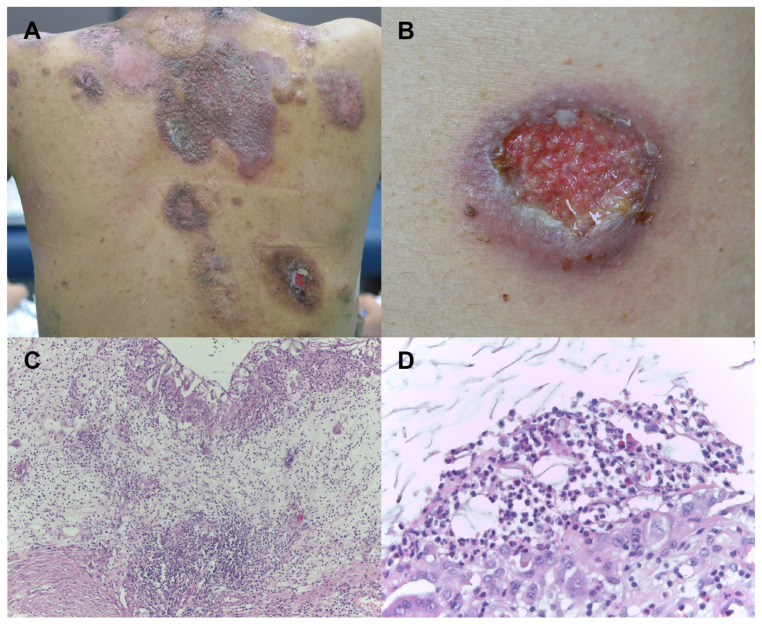
Skin findings of the patient. (**A**) Multiple violaceous erosive and ulcerative patches were scattered over the body. (**B**) Ulcerative patch with undermined violaceous border was observed. (**C**) Histopathologic examination showed intense neutrophilic infiltration with epidermal necrosis and dermal edema (H&E, ×100). (**D**) Neutrophilic and histiocytic infiltration was noted. (H&E, ×400).

A 36-year-old man had recurrent ulcerative skin lesions and was diagnosed to have cutaneous PG. Ulcerative patches with violaceous undermined border were observed over the trunk and extremities (Figure 1). Tissue cultures were negative and pathologic examination showed intense neutrophilic infiltration with tissue necrosis (Figure 1). Diagnosis of PG was made depending on the diagnostic criteria suggested by the Delphi Consensus of International Experts [5], meeting one major criteria (biopsy of ulcer edge demonstrating a neutrophilic infiltrate) and five minor criteria (exclusion of infection; history of papule, pustule, or vesicle that rapidly ulcerated; peripheral erythema, undermining border, and tenderness at site of ulceration; multiple ulcerations; and decrease in ulcer size within 1 month of initiation of immunosuppressive medications).Systemic evaluation revealed no associated inflammatory diseases or cancers. He was under prolonged immunosuppressive treatment with prednisolone (15 mg/day), cyclophosphamide (150 mg/d), and mycophenolate mofetil (1500 mg/d). 

**Figure 2:**
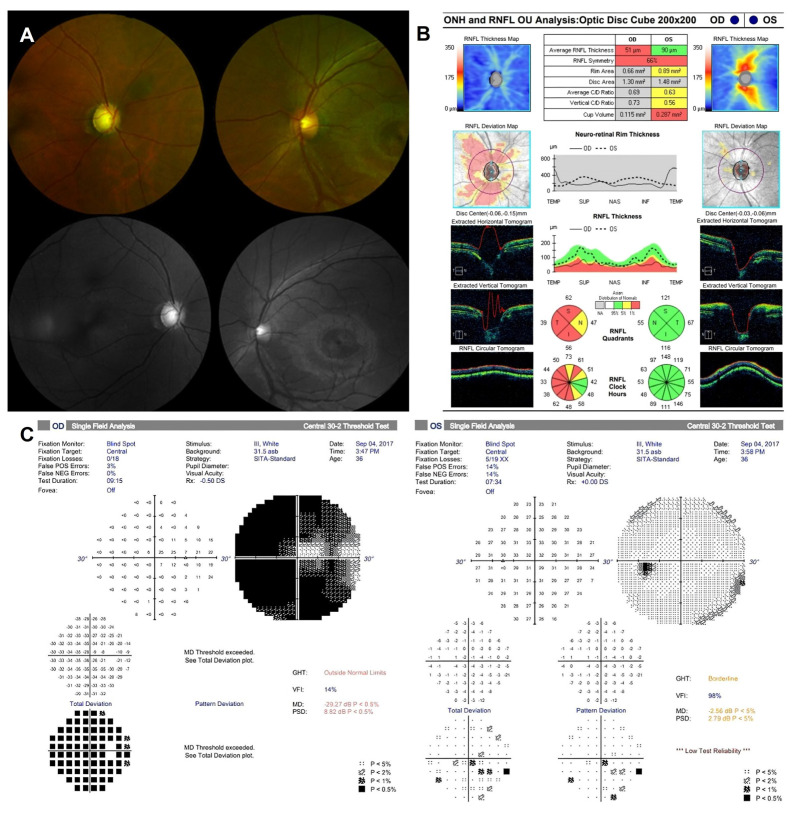
Ophthalmic images of the patient at the initial visit. (**A**) Fundus photography showed glaucomatous cupping of optic nerve head (ONH) in the right eye and normal ONH in the left eye. (**B**) Optical coherence tomography showed the thinning of retinal nerve fiber layer (RNFL) in all four quadrants of the right eye and the normal range of RNFL in the left eye. (**C**) Humphrey 30-2 visual field revealed of visual field narrowing in the right eye compared to the left eye.

**Figure 3:**
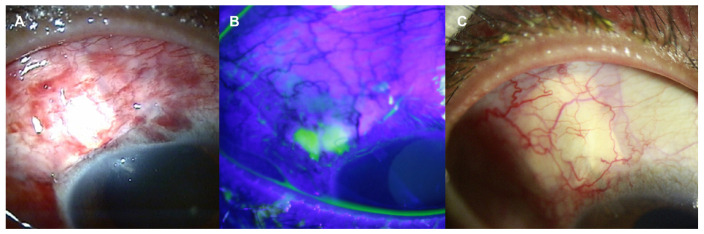
Slit-lamp photos of the right eye after Ahmed glaucoma valve implantation with donor scleral patch graft. (**A**) Conjunctiva was well approximated without dehiscence after 1 month of surgery. (**B**) At postoperative 2 months, however, mild conjunctival necrosis at the suture site developed. The necrotic area was highlighted by fluorescein stain. (**C**) It was recovered at postoperative 4 months follow up.

After 3 years of immunosuppressive medication, redness and pain developed in the right eye, and visual acuity was also decreased in the right eye. Complete ophthalmic examinations, including best-corrected visual acuity (BCVA) test, slit-lamp biomicroscopy, intraocular pressure (IOP) measurement, dilated fundus examination, optical coherent tomography (OCT), and visual field examination were performed. The BCVA was 20/20 in the left eye, but it was decreased to 20/50 in the right eye. The IOP measured by Goldmann applanation tonometer (GAT) was 16 mmHg in the left eye, but it was increased to 47 mmHg in the right eye (normal range, 10–21 mmHg). The slit-lamp examination revealed a mild corneal epithelial edema in the right eye. Fundus examination of the right eye showed an increased cup-disc ratio of 0.9 relative to 0.4 of the left eye. OCT showed thinning of the retinal nerve fiber layer (RNFL) in all four quadrants of the right eye (average, 51 µm) compared with the left eye (average, 90 µm). Perimetry by a Humphrey visual field analyzer (30-2 threshold program) revealed a narrowing of the visual field in the right eye when compared to that in the left eye (Figure 2). Based on the findings, SIG in the right eye was diagnosed and maximal medical therapy was attempted, encompassing oral and intravenous hyperosmotic agents and topical agents such as dorzolamide, timolol, brimonidine tartrate, and latanoprost. Even though the BCVA was restored to 20/25, and the pain disappeared, high IOP was maintained at 36 mmHg by GAT. Surgical management was decided and aqueous shunt surgery of AGV implantation was chosen instead of trabeculectomy to minimize the surgical trauma to the patient. In addition, donor scleral patch graft was adopted instead of scleral flap to avoid the incision into the sclera. The surgery was successful with a remarkable postoperative recovery of IOP to 10 mmHg by GAT. At postoperative 2 months, however, slit lamp examination revealed mild conjunctival necrosis at the suture site, which was recovered at postoperative 4 months follow-up (Figure 3). 

**Figure 4:**
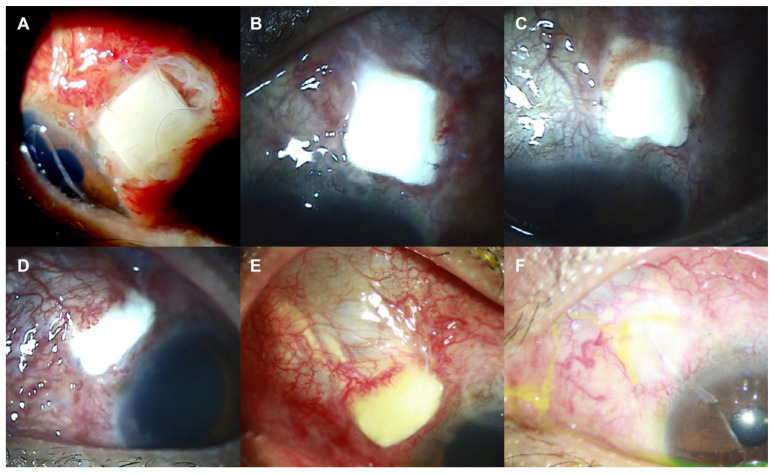
Slit-lamp photos of the left eye after initial (**A**–**C**) and secondary (**D**–**F**) Ahmed glaucoma valve (AGV) implantation with donor scleral patch graft. (**A**–**C**) Conjunctival necrosis with scleral patch exposure was observed at postoperative 1 (**A**), 2 (**B**), and 3 (**C**) months after initial AGV implantation. (D-F) Conjunctival necrosis with scleral patch exposure was detected again at postoperative 1 week (**A**) and 1 month (**B**) after secondary AGV implantation. (**F**) Conjunctival defects were healed at postoperative 4 months along with the conjunctival thinning.

**Figure 5:**
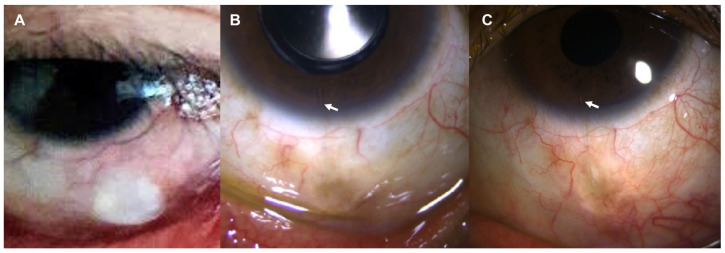
Slit-lamp photos of the left eye after the XEN^®^ Gel Stent implantation. (**A**) One month after the implantation, conjunctival bleb was prominent with intact conjunctiva. (**B**) At postoperative 1 year, conjunctival bleb was well maintained, which is still intact 3 years after the surgery (**C**). White arrow indicates XEN^®^ Gel Stent in the anterior chamber.

Three months after the treatment of SIG in the right eye, IOP elevation was also noticed in the left eye. Despite maximal medical therapy, IOP was 36 mmHg by GAT, and a mild corneal epithelial edema was observed in the left eye. Under the diagnosis of SIG in the left eye, the same operation of AGV implantation with donor scleral patch graft was performed. The initial surgical outcome was favorable with the postoperative IOP of 7 mmHg by GAT. However, after 2 months, severe eye pain with re-elevation of the IOP ranging between 32 and 48 mmHg was detected. The slit-lamp examination revealed the necrosis of conjunctiva with the exposure of scleral patch in the superotemporal quadrant (Figure 4). Conjunctival necrosis was not restored despite the conservative management. Because of the uncontrolled IOP, secondary AGV implantation with donor scleral patch graft was conducted in the superonasal quadrant, and conjunctival dehiscence was repaired with debridement of the previous scleral patch in the superotemporal quadrant. The initial outcome after the second surgery seemed to be favorable with remarkable postoperative recovery of IOP to 7 mmHg by GAT; however, conjunctival dehiscence of the superonasal quadrant was observed again 1 week after the surgery (Figure 4). Conjunctival dehiscence was managed with conservative treatment but persisted beyond the regular healing time frame, resulting in ocular pain in the patient, so it was considered as the ocular involvement of PG. At that time, his skin lesions were also aggravated with multiple enlarging hemorrhagic ulcers. Four months after the second surgery on the left eye, the conjunctival dehiscence was finally healed, leaving conjunctival thinning, but the encapsulated bleb formation led to the re-rise of IOP to 34 mmHg by GAT. After the repetitive failure of AGV implantation in the left eye, MIGS was adopted, and a collagen implant XEN^®^ Gel Stent (AqueSys, CA, USA) was placed in the inferior quadrant with subconjunctival injection of 0.1 mL of 0.2 mg/mL mitomycin C (Figure 5). At 1 month after the XEN^®^ Gel Stent implantation, the conjunctival bleb was prominent without conjunctival necrosis, and the IOP was successfully decreased to between 8 and 12 mmHg by GAT in the left eye. At postoperative 1 year, the BCVA was 20/32 in the right eye and 20/20 in the left eye. IOP was maintained at 13 mmHg in the right eye and 11 mmHg in the left eye by GAT without conjunctival necrosis. During 3 years of follow-up, IOP has been well controlled in both eyes.

## 3. Discussion

Even though there are few epidemiologic data on PG, the incidence of PG is relatively low, and it is estimated to 3–10 cases per million people per year [6]. Ocular involvement of PG is a rare clinical variant and has variable clinical presentations. A recent systematic review identified 34 published case reports of ocular PG. Some cases may be cutaneous, involving the periocular tissue, and others may be extracutaneous, involving the eye itself. The most common presenting sign of ocular PG is ulceration and often occurs at sites of trauma [7]. The phenomenon of pathergy, which means the development of a new inflammatory lesion at the site of trauma, is commonly described in the patients with PG [1]. In this case, the patient presented conjunctival necrosis after ocular surgery. 

There are currently no definitive treatment guidelines for PG, and the approach to treatment is typically based on reducing inflammation through immunomodulation. Systemic glucocorticoids are typically considered first-line therapy for severe cases of PG, as the disease can progress rapidly. Fast-acting immunosuppressants such as corticosteroids or cyclosporine are often more effective in these cases. Because long-term steroid therapy is associated with significant adverse effects, other steroid-sparing adjunctive agents can be used. The use of other immunosuppressive agents, including methotrexate, amino phenolic acid, and azathioprine, have been reported, and biologic drugs such as anti-tumor necrosis factor agents have shown promise as second-line treatments [1].

In susceptible individuals, the use of steroids can lead to a clinical condition such as primary open angle glaucoma. Approximately 5% of the population are considered high-steroid responders and may experience an increase in IOP. Studies have shown that there are several risk factors for the possibility of high-response such as personal or family history of primary open angle glaucoma, old age or age less than 6 years, type 1 diabetes mellitus, high myopia, and connective tissue disorders, especially rheumatoid arthritis in men [8]. The patient had no known risk factors.

The most common routes of steroids inducing glaucoma are through topical, intraocular, or periocular administration, as well as systemic steroid use, skin application, intranasal application, or inhalation. The incidence of SIG following systemic therapies is less common than following topical administration, and while an acute response can occur with intensive systemic steroid therapy, the IOP response often takes years to develop [8].

In general, surgical intervention is avoided in patients with PG due to the risk of a pathergic reaction [9]. However, some experts have suggested that after the disease has been stabilized with immunosuppressants, surgical management may be considered as a means of improving wound healing and reducing morbidity [10,11,12].

Our patient, with SIG in both eyes due to prolonged use of systemic corticosteroid for PG, demanded IOP-lowering surgery to prevent the progression of glaucoma. AGV implantation with donor scleral patch graft was selected to reduce surgical trauma in the patient, and the outcome was successful in the right eye. However, the same surgery was complicated with prolonged conjunctival dehiscence which lasted for months despite conservative management in the left eye. Based on the observation that cutaneous lesions were also worsened at the time of surgery on the left eye, the disease activity might have affected the pathergic reaction in the patient.

Due to the lack of available literature on the optimal surgical approach for treating glaucoma in patients with PG, determining the appropriate surgical options was challenging. However, based on the invasiveness of surgical options, MIGS would be the first choice, followed by tube surgery as the second preference, and cyclo-destructive procedures as the third option, in order of preference. However, since MIGS was not widely available at the time of the first surgery, a tube surgery was selected as the initial option.

When considering a tube surgery, using a scleral flap could result in a large incision that may lead to necrosis. As a result, a donor scleral patch graft was used in this case. Another potential option could be a scleral tunnel with amniotic membrane.

AGV implantation is an effective surgical option in the management of refractory glaucoma; however, several postoperative complications have been reported, including tube exposure, hypotony, encapsulated bleb, and others [13,14]. The surgery in the left eye eventually failed with an encapsulated bleb formation in this patient. Having multiple glaucoma surgeries in the same eye can make the ocular surface susceptible to surface disorders, fibrosis, and cicatricial changes, leading to chronic inflammation, moderate to severe xerosis, and other related issues. The presence of persistent surface inflammation can also contribute to the failure of tube and filtering surgeries, as well as conjunctival and scleral necrosis. Therefore, a cyclo-destructive procedure would have been considered as a potential option if the tube surgery were to fail or not result in an adequate response.

MIGS have been developed as safer and less traumatic surgical interventions for patients with mild to moderate glaucoma or who are intolerant to standard medical therapy. MIGS are generally defined as surgical procedures with an ab interno approach, minimal trauma with very little or no scleral dissection, minimal or no conjunctival manipulation, good safety profile, and rapid recovery [15]. The XEN^®^ Gel Stent is a subconjunctival microinvasive glaucoma surgical device developed with the aim of improving the predictability and safety profile of bleb forming glaucoma surgical procedures. The stent is a hydrophilic tube composed of a porcine gel cross-linked with glutaraldehyde with good stability and biocompatibility with minimal tissue reaction. This device has demonstrated promising outcomes with fewer risks compared to traditional surgeries [16]. The XEN^®^ Gel Stent can be indicated for the management of refractory glaucoma where previous surgical treatment has failed or in patients with primary open angle glaucoma who are unresponsive to maximum tolerated medical therapy. Grover et al. [17] reported the efficacy and safety of the XEN^®^ Gel Stent in patients with refractory glaucoma who failed prior filtering/cyclo-destructive procedure or had uncontrolled IOP on maximum tolerated medical therapy. At 12 months, 76.3% of patients achieved 20% or more IOP reduction from baseline on the same or fewer medications, and the mean diurnal IOP reduction from baseline was −6.4 mmHg [17]. The refractory glaucoma in the left eye of the patient was finally managed with MIGS and maintained the normal range of IOP for 3 years postoperative with no surgical complications.

## 4. Conclusions

The authors experienced a case of refractory SIG in a patient with PG in which ocular involvement of conjunctival necrosis was observed after AGV implantation and whose glaucoma was successfully managed with MIGS. This is the first case report describing the surgical management of SIG in a patient with PG. Ophthalmic surgery can be complicated in patients with PG, and the surgical option should be selected prudently to minimize surgical trauma. MIGS, as a minimally invasive surgical technique, could offer an advantage for patients with PG.

## Data Availability

Not applicable.
